# Brief research report: ETS-1 blockade increases ICAM-1 expression in activated human retinal endothelial cells

**DOI:** 10.3389/fopht.2024.1384428

**Published:** 2024-04-16

**Authors:** Alwin Chun Rong Tan, Yuefang Ma, Binoy Appukuttan, Karen Lower, Amanda L. Lumsden, Michael Z. Michael, Justine R. Smith, Liam M. Ashander

**Affiliations:** Flinders University College of Medicine and Public Health, Flinders University, Adelaide, SA, Australia

**Keywords:** human, uveitis, retinal endothelial cell, TNF-α, IL-1β, ICAM-1, ETS-1

## Abstract

Intercellular adhesion molecule 1 (ICAM-1) is a central cell adhesion molecule for retinal transendothelial migration of the leukocytes in non-infectious posterior uveitis. Inhibiting *ICAM1* gene transcription reduces induction of ICAM-1 in inflamed retinal endothelium. Based on published literature implicating transcription factor ETS-1 as an activator of *ICAM1* gene transcription, we investigated the effect of ETS-1 blockade on ICAM-1 levels in cytokine-stimulated human retinal endothelial cells. We first examined *ICAM1* and *ETS1* transcript expression in human retinal endothelial cells exposed to tumor necrosis factor-alpha (TNF-α) or interleukin-1beta (IL-1β). *ICAM1* and *ETS1* transcripts were increased in parallel in primary human retinal endothelial cell isolates (n = 5) after a 4-hour stimulation with TNF-α or IL-1β (p ≤ 0.012 and ≤ 0.032, respectively). We then assessed the effect of ETS-1 blockade by small interfering (si)RNA on cellular *ICAM1* transcript and membrane-bound ICAM-1 protein. *ETS1* transcript was reduced by greater than 90% in cytokine-stimulated and non-stimulated human retinal endothelial cell monolayers following a 48-hour treatment with two ETS-1-targeted siRNA, in comparison to negative control non-targeted siRNA (p ≤ 0.0002). The ETS-1 blockade did not reduce *ICAM1* transcript expression nor levels of membrane-bound ICAM-1 protein, rather it increased both for a majority of siRNA-treatment and cytokine-stimulation conditions (p ≤ 0.018 and ≤ 0.004, respectively). These unexpected findings indicate that ETS-1 blockade increases ICAM-1 transcript and protein levels in human retinal endothelial cells. Thus ETS-1-targeting would be expected to promote rather than inhibit retinal transendothelial migration of leukocytes in non-infectious posterior uveitis.

## 1 Introduction

Non-infectious posterior uveitis is an uncommon, but sight-threatening group of autoimmune and autoinflammatory eye diseases (1). Anti-metabolites and calcineurin inhibitors are typically the first-line immunomodulatory drugs used to treat the inflammation. However, these drugs are ineffective in achieving disease control in up to 50% of patients, and there is continuing interest in the identification and evaluation of biologic therapeutic approaches that target disease mechanisms (2). A central pathogenic event in any form of non-infectious posterior uveitis is the migration of leukocytes from the circulation into the posterior eye, mediated by cell adhesion molecules (3).

Intercellular adhesion molecule 1 (ICAM-1), also known as CD54, is a member of the immunoglobulin (Ig) superfamily that is widely expressed constitutively across diverse cell populations, with homeostatic functions that include leukocyte trafficking for immune surveillance, formation of the immune synapse and wound healing (4). During pathological tissue inflammation, induction of ICAM-1 on the local vascular endothelium by inflammatory cytokines is a key molecular mechanism that underpins leukocyte extravasation (5). Our previously published work has demonstrated that ICAM-1 blockade reduces human retinal endothelial transmigration of leukocytes implicated in the initiation of non-infectious posterior uveitis: Th1 cells, Th17 cells and B cells (6, 7). We have further shown that targeting transcription of the gene that encodes ICAM-1 – *ICAM1* – can reduce induction of the protein on the retinal endothelium without impacting constitutive expression (8). Thus, drugging gene transcription has the potential to limit ICAM-1 involvement in pathological inflammation, while preserving its homeostatic functions.

The *ICAM1* gene promoter contains multiple binding sites for the transcription factor, ETS proto-oncogene 1, transcription factor (ETS-1, encoded by the *ETS1* gene) (9). Reduced skin ETS-1 and ICAM-1 have been linked in patients with vitiligo, and silencing of ETS-1 using small-interfering (si)RNA reduces levels of *ICAM1* transcript and ICAM-1 protein in cultured human melanocytes (10). Consistently, ETS-1 overexpression increases ICAM-1 levels in the H460 non-small cell lung cancer cell line, associated with migratory ability and invasiveness (11). Cooperative upregulation of *ICAM1* gene transcription by ETS-1 and autoimmune regulator (AIRE) in human oral squamous cell carcinoma cells, and by ETS-1 and signal transducer and activator of transcription 1 (STAT 1) in human HEK293 and monkey COS-1 kidney cell lines are also reported (12, 13).

Based on these observations, we investigated ETS-1 as a potential therapeutic target for limiting *ICAM1* gene transcription in the retinal endothelium following activation by inflammatory cytokines. Unexpectedly, we observed that ETS-1 blockade increased ICAM-1 levels in human retinal endothelial cells.

## 2 Methods

### 2.1 Human retinal endothelial cells

Primary human retinal endothelial cells were isolated from cadaveric donor eyes obtained from the Eye Bank of South Australia, under a protocol approved by the Southern Adelaide Clinical Human Research Ethics Committee (protocol 175.13), according to a previously published method (14). These cells were used in experiments at passage 2 to 3. One human retinal endothelial cell isolate was previously expanded in-house, by transduction with the pLXSN16E6E7 retroviral construct (gift of Dr. Denise Galloway, Fred Hutchinson Cancer Institute, Seattle, WA) (3). Human retinal endothelial cells were cultured in modified MCDB-131 medium (Sigma Aldrich, St. Louis, MO) containing 10% heat-inactivated fetal bovine serum (Thermo Fisher Scientific-Gibco, Auckland, New Zealand) and EGM-2 SingleQuots Kit (Lonza-Clonetics, Walkersville, MD) omitting the hydrocortisone and gentamicin supplements, at 37°C and 5% CO_2_ in air.

### 2.2 Recombinant proteins and primary antibodies

Human recombinant tumor necrosis factor-alpha (TNF-α) and interleukin-1beta (IL-1β) were purchased from R&D Systems (Minneapolis, MN), and used at working concentrations of 5 or 10 ng/mL. Mouse monoclonal IgG2b_K_ anti-human ICAM-1 (clone LB-2) and isotype-matched negative control anti-dansyl (clone 27-35) antibodies were bought from BD Pharmingen (San Jose, CA), and used at a working concentration of 1 μg/mL.

### 2.3 Cell manipulations

For reverse transcription and relative quantitative real-time polymerase chain reaction (RT-qPCR), confluent monolayers of primary human retinal endothelial cells in 12-well plates (3.8 cm^2^ growth area) were treated with cytokine diluted in medium or fresh medium alone for 4 hours. For experiments involving ETS-1 silencing, sub-confluent monolayers of expanded human retinal endothelial cells were transfected with 12 pmol ETS-1-targeted Silencer Select siRNA (Thermo Fisher Scientific-Ambion, Foster City, CA; catalogue numbers: s4847 and s4848) or non-targeted siRNA (Silencer Select Negative Control No. 1, Thermo Fisher Scientific-Ambion) in 2 μL of Lipofectamine RNAiMAX Reagent (Thermo Fisher Scientific-Invitrogen, Vilnius, Lithuania) per well in 12-well plates (used for RNA extraction and reverse transcription), or 1 pmol ETS-1-targeted Silencer Select siRNA or non-targeted siRNA in 0.3 μL of Lipofectamine RNAiMAX Reagent per well in 96-well plates (0.32 cm^2^ growth area; used for the protein immunoassay). Following a 24-hour incubation, these treatments were refreshed with medium containing TNF-α, IL-1β, or no cytokine, and the cell monolayers were incubated for a further 24 hours prior to determination of levels of *ICAM1* transcript and ICAM-1 protein.

### 2.4 RNA extraction and reverse transcription

Total RNA was recovered from human retinal endothelial cells using either the GenElute Mammalian Total RNA Miniprep Kit (Merck-Sigma Aldrich, St. Louis, MO) or the RNeasy Mini Kit (Qiagen, Hilden, Germany). Cells were lysed in Lysis Solution for Total RNA or RLT Buffer, respectively, containing 1.4 mM 2-mercaptoethanol (Merck-Sigma Aldrich). Treatment with DNase I was included in all extractions. RNA concentration was determined using the Nanodrop 2000 spectrophotometer (Thermo Fisher Scientific, Wilmington, DE), and reverse transcription (RT) was performed using the iScript Reverse Transcription Supermix for RT-qPCR (Bio-Rad, Hercules, CA), with 100 ng of RNA yielding 20 μL of cDNA.

### 2.5 Quantitative real-time polymerase chain reaction

Relative quantification real-time polymerase chain reaction was performed using the CFX Connect Real-Time PCR Detection System (Bio-Rad). Each 20 μL PCR reaction contained, in addition to nuclease-free water, 2 μL of cDNA (diluted 10-fold), 4 to 10 μL of iQ SYBR Green Supermix or SSoAdvanced Universal SYBR Green Supermix (Bio-Rad), and 0.75 to 1.5 μL each of 10 μM forward and reverse primers. Cycling conditions consisted of: a pre-cycling incubation for 30 seconds or 5 minutes at 95°C; 40 cycles of denaturation for 30 seconds at 95°C, annealing for 30 seconds at 62°C, and extension for 30 seconds at 72°C. Melt curves were generated with 1-second holds at 0.5°C increments between 70°C and 95°C to confirm the amplification of a single PCR product. Amplicon sizes were confirmed by agarose gel electrophoresis. All samples were amplified in duplicate. Normalized expression was calculated by CFX Manager software v3.1 (Bio-Rad) using the geometric mean of the two reference genes, *GAPDH* (encoding glyceraldehyde 3-phosphate dehydrogenase) and *RPLP0* (encoding ribosomal protein lateral stalk subunit P0). Primer sequences were: *ETS1* forward 5’-GTGCTGACCTCAATAAGGA-3’, reverse 5’-GCTGATAAAAGACTGACAGGAT-3’ [expected product size: 134 bp (11)]; *ICAM1* forward- 5’-CCTTCCTCACCGTGTACTGG-3’, reverse 5’-AGCGTAGGGTAAGGTTCTTGC-3’ [expected product size: 90 bp (15)]; *GAPDH* forward 5’-AGCTGAACGGGAAGCTCACTGG-3’, reverse 5’-GGAGTGGGTGTCGCTGTTGAAGTC-3’ [expected product size: 209 bp (16)]; *RPLP0* forward 5’-GCAGCATCTACAACCCTGAA-3’, reverse 5’-GCAGATGGATCAGCCAAGAA-3’ [expected product size: 234 bp (17)].

### 2.6 Protein immunoassay

Membrane-bound ICAM-1 protein was measured by a previously published immunoassay (18). Cell monolayers were fixed in 1% w/v paraformaldehyde, blocked with 5% w/v skim milk in phosphate-buffered saline (PBS) and labelled with anti-ICAM-1 or negative control antibody in blocking solution for 45 minutes. Labelling was detected with 2.5 μg/mL Alexa Fluor 488-conjugated goat anti-mouse IgG antibody (Thermo Fisher Scientific-Molecular Probes, Eugene, OR) in blocking solution for 30 minutes and 300 nM 4′,6-diamidino-2-phenylindole (DAPI) (Merck-Sigma Aldrich) in PBS was applied for 5 minutes to counterstain nuclei. All incubations were performed at room temperature on an orbital shaker. Monolayer fluorescence was detected with the VICTOR X3 Multilabel Plate Reader (Perkin Elmer, Waltham, MA) with excitation and emission wavelengths of 485 and 535 nm (AlexaFluor 488 filter), plus excitation and emission wavelengths of 355 and 460 nm (DAPI filter), respectively. The DAPI filter readings were used to adjust for cell numbers across the monolayers. Mean background fluorescence was the average of readings from negative control antibody-labelled monolayers. True fluorescence was determined by subtracting mean background from readings of anti-ICAM-1 antibody-labelled monolayers.

### 2.7 Statistical analysis

Data analyses were performed using GraphPad Prism v9.0 (GraphPad Software, La Jolla, CA). Comparisons between two groups were made using the paired or unpaired two-tailed Student’s t-test. Comparisons across multiple groups were made using the ordinary two-way ANOVA with Sídák’s multiple comparison tests. In all testing, a p-value less than 0.05 was taken to indicate a statistically significant difference.

## 3 Results

Human retinal endothelial cell isolates were prepared from eyes of male and female cadaveric donors whose age ranged between 30 and 77 years at the time of death. The mean time from death to cell isolation was 25 hours.

**Figure 1:**
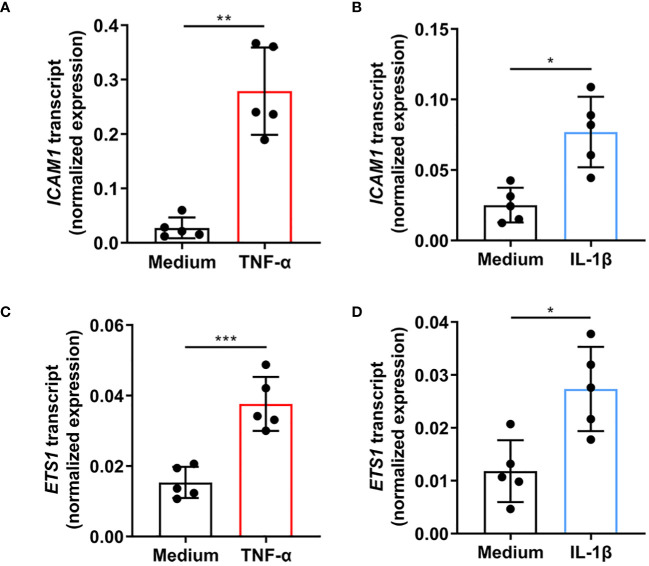
Expression of *ICAM1* and *ETS1* transcripts in human retinal endothelial cells treated with TNF-α and IL-1β. Graphs show expression of **(A, B)** *ICAM1* and **(C, D)** *ETS1* transcripts in primary retinal endothelial cell isolates from 5 human cadaveric donors following a 4-hour treatment with **(A, C)** TNF-α (10 ng/mL), **(B, D)** IL-1β (5 ng/mL), or fresh medium alone. Bars represent mean expression normalized to the expression of reference genes, *RPLP0* and *GAPDH*. Dots indicate individual donors (n = 5 donor/condition). Error bars show standard deviation. Data were analyzed by two-tailed paired t-test: *p < 0.05, **p < 0.01; ***p < 0.001.

Primary human retinal endothelial isolates were individually treated with TNF-α and IL-1β to evaluate the effect of cellular activation on ETS-1 and ICAM-1 co-expression. Cells isolated from 5 individual donors increased the expression of *ICAM1* transcript following a 4-hour treatment with TNF-α (p=0.003) or IL-1β (p=0.012) (**Figures 1A, B**). Cellular expression of *ETS1* transcript was also increased by the TNF-α (p=0.0004) and IL-1β (p=0.032) treatments (**Figures 1C, D**). These results indicate that in human retinal endothelial cells activated by inflammatory cytokines, there is a parallel induced expression of both ICAM-1 and transcription factor ETS-1.

**Figure 2:**
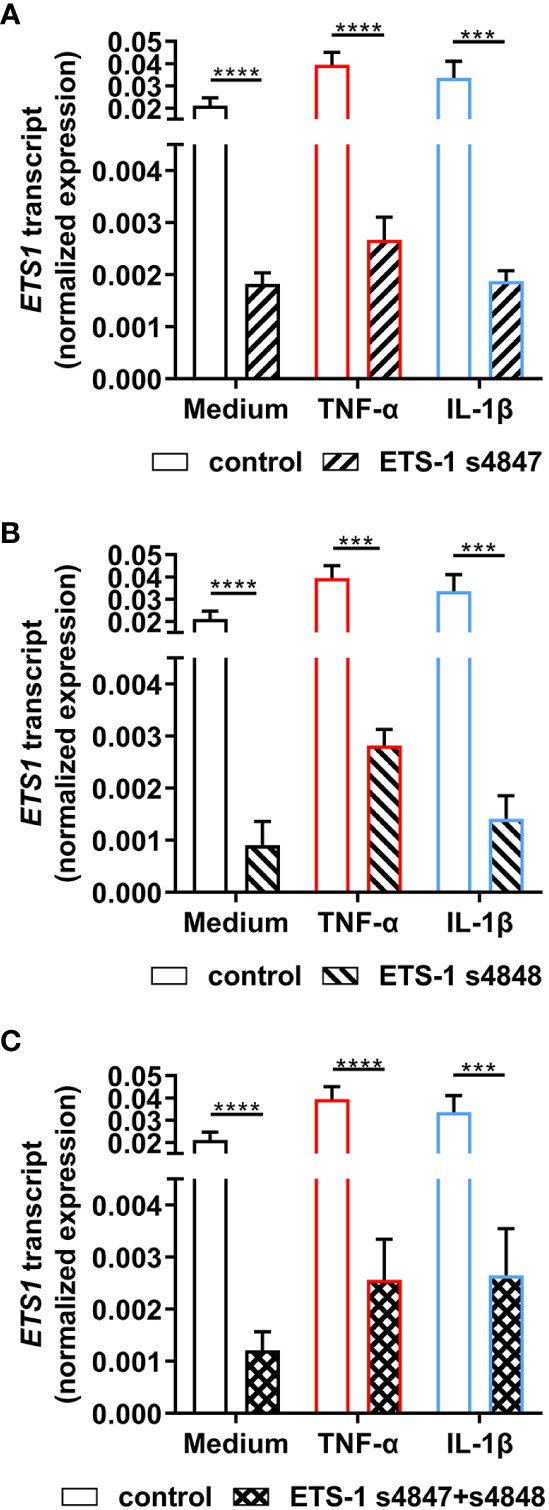
Effect of ETS-1-targeted siRNA on *ETS1* transcript expression in human retinal endothelial cells. Human retinal endothelial cells were transfected with ETS-1 siRNA s4847, s4848, s4847 plus s4848, or non-targeted control siRNA for 24 hours, and subsequently stimulated for another 24 hours with TNF-α (10 ng/mL), IL-1β (10 ng/mL) or fresh medium alone. Graphs show expression of *ETS1* transcript after transfection with **(A)** ETS-1 siRNA s4847, **(B)** ETS-1 siRNA s4848, and **(C)** ETS-1 siRNA s4847 plus s4848, versus the common non-targeted control siRNA. Bars represent mean expression normalized to the expression of reference genes, *RPLP0* and *GAPDH* (n = 3-4 monolayers/condition). Error bars show standard deviation. Data were analyzed by two-tailed unpaired t-test: ***p < 0.001; ****p < 0.0001.

To investigate the relationship between *ETS1* and *ICAM1* gene transcription in human retinal endothelial cells, siRNA knockdown of *ETS1* transcript was performed using two different targeted siRNA, with and without inflammatory cytokine treatment. To verify effective ETS-1 silencing, its transcript levels were evaluated by RT-qPCR. For these experiments, we used the expanded human retinal endothelial cell isolate since large numbers of cells were needed. Significant reduction in *ETS1* transcript expression was observed in both cytokine-treated and untreated human retinal endothelial cells 48 hours after transfection with individual targeted siRNA, as well as the combination, in comparison to non-targeted negative control siRNA: ETS-1 siRNA s4847, ≥ 91.4% mean reduction; ETS-1 siRNA s4848, ≥ 92.9% mean reduction; ETS-1 siRNA s4847 plus s4848, ≥ 92.1% mean reduction (p ≤ 0.0002) (**Figures 2A-C**).

**Figure 3:**
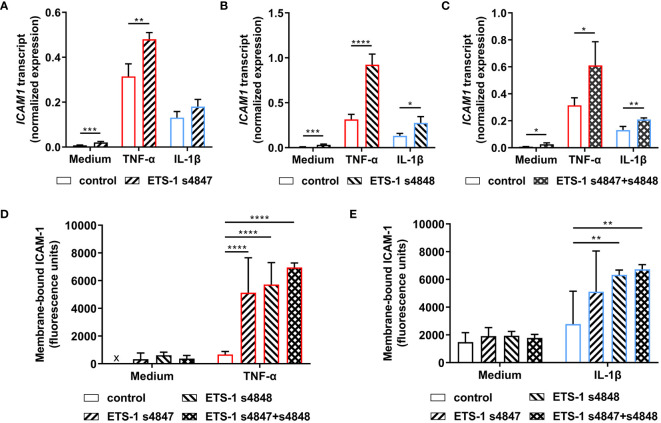
Impact of ETS-1 silencing on the levels of human retinal endothelial cell *ICAM1* transcript and membrane-bound ICAM-1 protein. Human retinal endothelial cells were transfected with ETS-1 siRNA s4847, s4848, s4847 plus s4848, or non-targeted control siRNA for 24 hours, and subsequently stimulated for another 24 hours with TNF-α (10 ng/mL), IL-1β (10 ng/mL) or fresh medium alone. **(A-C)** Graphs show expression of *ICAM1* transcript after transfection with **(A)** ETS-1 siRNA s4847, **(B)** ETS-1 siRNA s4848, and **(C)** ETS-1 siRNA s4847 plus s4848, versus the common non-targeted control siRNA. Bars represent mean expression normalized to the expression of reference genes, *RPLP0* and *GAPDH* (n = 3-4 monolayers/condition). Error bars show standard deviation. **(D, E)** Graph shows membrane-bound ICAM-1 protein after treatment with siRNA, followed by **(D)** TNF-α or fresh medium, and **(E)** IL-1β or fresh medium. Bars represent mean relative fluorescence of immunolabelled cell monolayers after corrections for background fluorescence and cell number (n = 4 cell monolayers/condition). Error bars show standard deviation. x = below detectable level. Data were analyzed by **(A-C)** two-tailed unpaired t-test and **(D, E)** two-way ANOVA, with Sídák post-hoc testing: *p < 0.05; **p<0.01; ***p < 0.001; ****p < 0.0001.

The effect of *ETS1* transcript knockdown on ICAM-1 expression by human retinal endothelial cells was first studied by RT-qPCR. Cellular transfection with ETS-1 siRNA resulted in an increased level of *ICAM1* transcript under unstimulated and cytokine-stimulated conditions (**Figures 3A-C**). This increase was statistically significant across all conditions when the *ETS-1* transcript was knocked down with siRNA s4848, or siRNA s4847 plus s4848, and for untreated and TNF-α-treated, but not IL-1β-treated, conditions when siRNA 4847 was used (p ≤ 0.018). These results show that robust knockdown of *ETS1* transcript is associated with an increase in the expression of *ICAM1* transcript in human retinal endothelial cells, both at baseline and following cytokine activation.

Given the unexpected observation that ETS-1 silencing increased *ICAM1* transcript expression in human retinal endothelial cells, the effect on expression of membrane-bound ICAM-1 protein was investigated in a protein immunoassay that quantified ICAM-1 expressed on the intact surface of cell monolayers. Cells transfected with individual or the combination of ETS-1 siRNAs and subsequently stimulated with cytokine demonstrated increased membrane-bound ICAM-1 in comparison to cells transfected with negative control siRNA (**Figures 3D, E**). Consistent with the observations made at transcript level, this increase was statistically significant for siRNA s4847, siRNA s4848, and the combination when cells were activated with TNF-α, and for siRNA s4848 and the combination, but not siRNA s4847, when cells were activated with IL-1β (p ≤ 0.004). Levels of membrane-bound ICAM-1 protein were consistently low in cells transfected with the control siRNA or ETS-1 siRNAs individually or in combination, when not exposed to an inflammatory cytokine. These results demonstrate that the increase in *ICAM1* transcript observed following ETS-1 blockade translates to an induction of membrane-bound ICAM-1 protein on activated human retinal endothelial cells.

## 4 Discussion

Drugging gene transcription is a therapeutic approach that has been explored recently, particularly in oncology (19). Induction of retinal endothelial ICAM-1 is a key event in the development of non-infectious posterior uveitis that is regulated at transcription (4). Based on published literature implicating transcription factor ETS-1 as an activator of *ICAM1* gene transcription (10–13), we considered it a promising drug target for blocking the induction of ICAM-1 during non-infectious posterior uveitis. We confirmed a parallel increase in *ICAM1* and *ETS1* gene expression in primary human retinal endothelial cells activated by exposure to either TNF-α or IL-1β. However, ETS-1 silencing in the cells resulted in increased, rather than decreased, ICAM-1 expression in this cell population, which we demonstrated at both transcript and protein level, and using two different siRNAs.

While there are no previous reports of ETS-1 repressing *ICAM1* gene expression, there are examples of ETS-1 repressing the activity of other genes. ETS-1 disrupts promoter interactions of two other transcription factors, NF-κB and CREB, to limit IL-1β-induced *MUC5AC* gene expression in human airway epithelium (20). Site-directed promoter site mutagenesis and siRNA transcription factor silencing studies in HEK293T human embryonic kidney cells have demonstrated that ETS-1 and transcription factor GFI1 cooperate to repress the *BAX* gene promoter (21). Additionally, ETS-1 directly represses transcription of the *PRDM1* gene, which encodes B lymphocyte-induced maturation protein 1, in human T helper 1 cells (22). A bioinformatics investigation of the *Ets1*-knockout versus wild-type mouse B cell transcriptomes revealed that 276 of the 484 differentially expressed gene transcripts were increased when ETS-1 was deleted, suggesting widespread transcriptional repression by ETS-1 in this cell population (23).

ETS family transcription factors regulate, and are regulated by, a large number of microRNAs (miRNAs) (24). This raises the possibility that ETS-1 regulation of miRNA might provide another explanation for our findings. Intriguingly, ETS-1 and ETS-2 cooperatively bind the *EGFL7* gene promoter to induce expression of miR-126, which targets vascular cell adhesion molecule 1 (VCAM-1); ETS-1/ETS-2 silencing decreases miR-126 expression, and increases VCAM-1 expression in human umbilical vein endothelial cells after stimulation with TNF-α (25). A study of IL-6-treated EA.hy926 vascular endothelial cells has linked miR-126 and ICAM-1 expression, although the mechanism remains unclear (26). Work involving melanoma cell lines suggests that in certain contexts ETS-1 up-regulates miR-222 (27), a miRNA that reduces ICAM-1 protein in TNF-α-treated human coronary artery endothelial cells (28).

There are multiple ETS-1 protein isoforms (29). Thus, some differences that we observed in the effects of ETS-1 silencing on ICAM-1 levels with the two siRNA under different treatment conditions might relate to effects of individual isoforms on *ICAM1* gene regulation in human retinal endothelial cells. Both siRNAs used in the study target the major ETS-1 isoform, alternatively referred to as p51 or p54, as well as minor isoform p42, which has relatively high DNA binding affinity. The ETS-1 siRNA s4847 also targets another minor isoform, p27, which has augmented autoinhibitory capacity (30).

In conclusion, we have showed that *in vitro* ETS-1 blockade in human retinal endothelial cell monolayers is associated with an increased level of ICAM-1. ICAM-1 protein expressed on the retinal endothelial cell membrane mediates transendothelial migration of leukocytes (6, 7). Thus ETS-1 blockade would be anticipated to promote leukocyte binding to and extravasation across the retinal endothelium. Our findings suggest ETS-1 blockade would promote rather than inhibit retinal transendothelial migration of leukocytes, and thus imply that ETS-1 is unlikely to be a viable drug target for non-infectious posterior uveitis. However, *in vitro* functional experiments and/or *in vivo* studies would be important to confirm this conclusion.

## Data availability statement

The original contributions presented in the study are included in the article/supplementary material. Further inquiries can be directed to the corresponding author.

## Ethics statement

The studies using primary human retinal cells were approved by the Southern Adelaide Clinical Human Research Ethics Committee (protocol 175.13). The studies were conducted in accordance with the local legislation and institutional requirements. Written informed consent for participation did not apply because the participants were deceased persons. Next-of-kin gave consent for the research use of eye tissue that would otherwise be discarded after removal of corneas for use in corneal transplantation surgeries.

## Author contributions

AT: Formal analysis, Investigation, Visualization, Writing – original draft. YM: Investigation, Writing – review & editing. BA: Conceptualization, Methodology, Supervision, Writing – review & editing. KL: Writing – review & editing, Conceptualization, Methodology, Supervision. AL: Writing – review & editing, Methodology, Supervision. MM: Writing – review & editing, Conceptualization, Methodology, Supervision. JS: Writing – original draft, Conceptualization, Formal analysis, Funding acquisition, Methodology, Supervision, Visualization. LA: Writing – original draft, Conceptualization, Formal analysis, Methodology, Supervision, Visualization.
